# Variations in the Pattern of the Deep Palmar Arch of the Hand and Its Surgical Importance

**DOI:** 10.7759/cureus.20873

**Published:** 2022-01-02

**Authors:** Pooja Dawani, Anita Mahajan, Neelam Vasudeva, Sabita Mishra

**Affiliations:** 1 Department of Anatomy, Subharti Medical College, Meerut, IND; 2 Department of Anatomy, Maulana Azad Medical College, New Delhi, IND

**Keywords:** morphometry, arterial variations, ulnar artery, radial artery, deep palmar arch

## Abstract

Background

The deep palmar arch is formed by anastomosis of the continuation of the radial artery with the deep palmar branch of the ulnar artery. With recent advances in microsurgical techniques for vascular repair, the knowledge of variations in the arteries of the hand, as well as the caliber of these arteries, has become more important for surgeons. Additionally, radial artery harvesting for myocardial revascularization is being performed nowadays, for which collateral circulation in the hand through the palmar arches is a prerequisite. Therefore, this study was conducted to study the patterns of the deep palmar arch and perform the morphometry of the arch.

Methodology

In this study, 30 hands (16 right and 14 left) from formalin-fixed adult human cadavers were dissected to observe the completeness, formation, and branching pattern of the deep palmar arch. The length of the arch was measured using a thread and scale. The diameters of the forming arteries and branches of the arch were measured at their origin using a digital vernier caliper.

Results

All deep palmar arches were complete. The arches were classified into two types based on whether the superior or inferior deep palmar branch of the ulnar artery completed the arch. Another classification was based on the interosseous space through which the radial artery or its branch entered the palmar region to complete the deep palmar arch. The length of the arch was 4.2 ± 0.47 cm on the right side and 4.0 ± 0.6 cm on the left side. The diameters of the deep palmar branch of the radial and ulnar arteries at their origin were 4.02 ± 0.48 mm and 1.90 ± 0.36 mm, respectively. No significant difference was found between the right and left sides.

Conclusions

The anastomosis was found between radial and ulnar arteries in all cases of the deep palmar arch. Therefore, it can be safe to sacrifice the radial artery in procedures such as radial artery harvesting and radial artery flap transfer. The knowledge of variations and morphometry of the arch will facilitate vascular repair surgeries on hands.

## Introduction

The deep palmar arch is important for the blood supply of the palm and fingers. It is usually formed by anastomosis between the continuation of the main radial artery and the deep palmar branch of the ulnar artery. When there is no anastomosis, the arch is said to be incomplete. Through its distal, recurrent, and perforating branches, the deep palmar arch ensures the blood supply of the hand [1].

The radial artery is the main artery contributing to the deep palmar arch. The damage to the radial artery in invasive procedures affects the blood flow through the palmar arches. Radial artery catheterization and radial artery harvesting for myocardial revascularization are commonly performed procedures. Transradial access is preferred over transfemoral access for coronary angiography and interventions in patients with acute coronary syndromes [2]. Moreover, transradial access is also used for cerebral angiography and neuroendovascular interventions [3]. However, transradial access for coronary and cerebral interventions has adverse effects, including radial artery occlusion, injury, spasm, perforation, pseudoaneurysm, or hematoma formation [4]. These complications may lead to compromised blood flow to the hand. This occurs in the absence of viable collateral circulation through the ulnar artery, which occurs in cases of incomplete deep palmar arches. There have been reports of ischemia of the hand after radial artery removal for coronary artery bypass surgery [5]. Thus, it is imperative to understand the variations of the deep palmar arch.

In distal transradial access, the radial artery is approached in the anatomical snuffbox. This approach is advantageous over the traditional transradial access as the superficial palmar branch of the radial artery arises proximal to the access site [6]. However, blood flow to the deep palmar arch and dorsal metacarpal arteries is jeopardized if occlusion of the radial artery occurs in the anatomical snuffbox. In such situations, the interossei are likely to be affected as they are supplied by the deep palmar arch and dorsal metacarpal arteries and do not receive supply from the superficial palmar arch [7].

With recent advances in microsurgical techniques for vascular repair, the knowledge of variations in arteries, as well as the caliber of these arteries, has become more important for surgeons. The pedicled interossei and lumbrical muscle flaps are used in reconstructive surgery of the hand. The lumbricals are supplied by branches from both the superficial and deep palmar arches, and interossei are supplied by the deep palmar arch and dorsal metacarpal arteries [7].

Given the importance of the deep palmar arch in the blood supply of the hand and the contribution of the radial artery in its formation, this study was conducted to study the morphology and morphometry of the deep palmar arch and its branches. This information will help surgeons to plan and perform microsurgeries in this region and propose newer and safer surgical procedures.

## Materials and methods

The present study was conducted in the Department of Anatomy, Maulana Azad Medical College, New Delhi from September 2015 to May 2017 after obtaining approval from the Institutional Ethics Committee (Approval no. 11/IEC/MAMC/2015/317). A total of 30 hands (16 right and 14 left) from formalin-fixed adult human cadavers were dissected. Normal as well as variant patterns in the arteries contributing to the arch, completeness of the arch, and the branching patterns were observed and noted, and their percentages were calculated.

The length of the arterial arch was measured using a thread and a scale. With the help of digital vernier calipers, the diameters of the forming arteries of the arch were measured at their origin. In addition, the diameters of palmar metacarpal branches were measured at their origin. The mean and standard deviation of the parameters were calculated. The unpaired t-test was applied to compare the values of the right and the left sides. A P-value of less than 0.05 was considered significant. Statistical analysis was done using SPSS version 20 (IBM Corp, Armonk, NY, USA).

## Results

**Figure 1:**
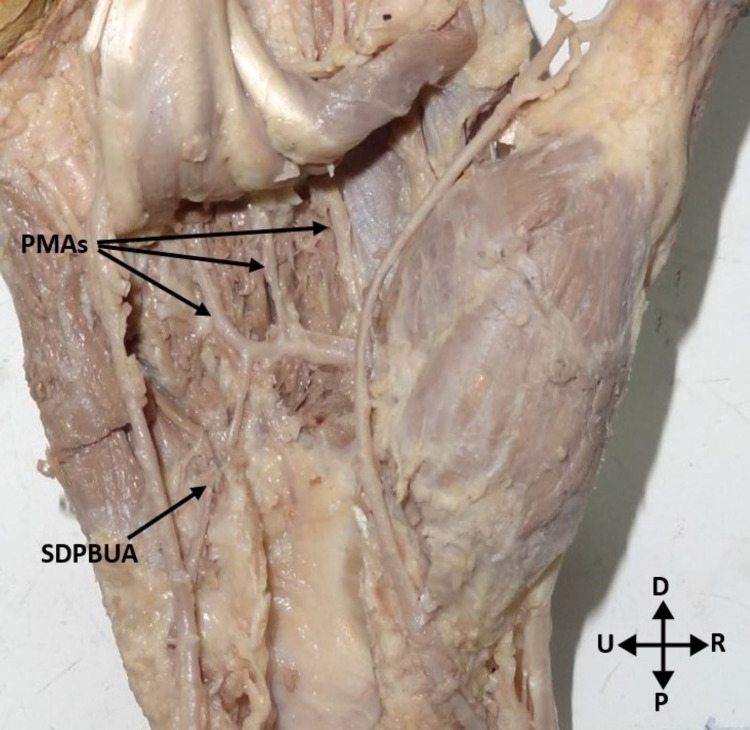
Deep palmar arch formed by the continuation of the radial artery and the superior deep palmar branch of the ulnar artery. PMAs: palmar metacarpal arteries; SDPBUA: superior deep palmar branch of the ulnar artery; P: proximal; D: distal; R: radial; U: ulnar

**Figure 2:**
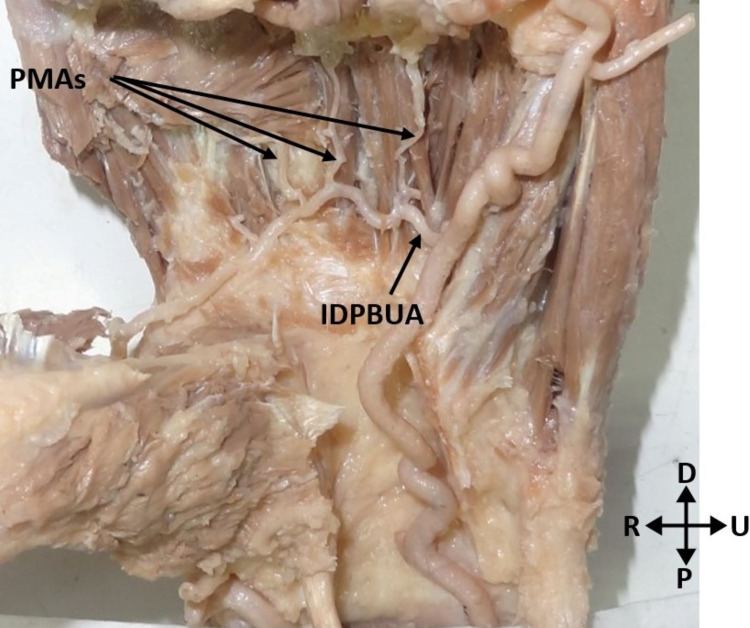
Deep palmar arch formed by the radial artery and the inferior deep branch of the ulnar artery. PMAs: palmar metacarpal arteries; IDPBUA: inferior deep palmar branch of the ulnar artery; P: proximal; D: distal; R: radial; U: ulnar

All deep palmar arches were complete, implying that there was anastomosis between the radial artery and the deep palmar branch of the ulnar artery. The deep palmar arch was classified into two types based on the type of the deep palmar branch of the ulnar artery completing the arch. In the Type 1 arch, the superior deep branch of the ulnar artery was anastomosed with the radial artery to complete the deep palmar arch. It was found in three (10%) hands (Figure 1). In the Type 2 arch, the inferior deep branch of the ulnar artery was anastomosed with the radial artery to complete the deep palmar arch. It was found in 27 (90%) hands (Figure 2).

**Figure 3:**
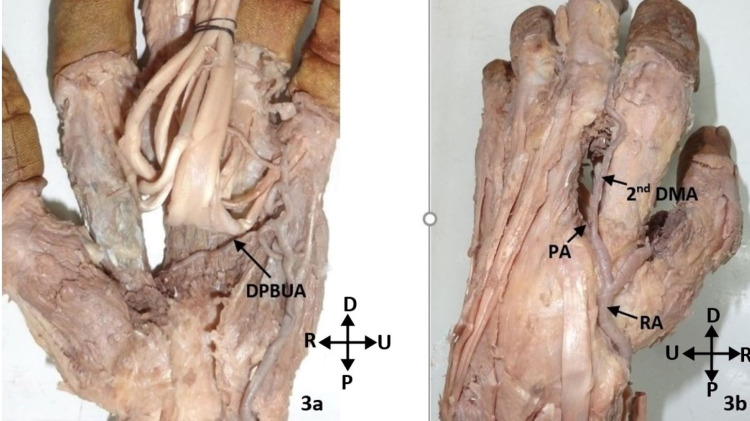
A: Deep palmar arch formed from the inferior deep branch of the ulnar artery and completed by a perforating artery from the second dorsal metacarpal artery. B: Dorsal view of the hand shown in A. DPBUA: deep palmar branch of the ulnar artery; DMA: dorsal metacarpal artery; PA: perforating artery; RA: radial artery; P: proximal; D: distal; R: radial; U: ulnar

Another classification of the deep palmar arch was employed based on the interosseous space through which the radial artery or its branch entered the palmar region to complete the deep palmar arch. In this classification, there were two types. In the Type 1 arch, the radial artery passed dorsally just distal to the radial styloid process, then passing deep to the extensor tendons, it entered the first intermetacarpal space to reach the palmar region. This type was found in 29 (96.7%) hands. The Type 2 arch included only one specimen (3.3%). In this hand, the second dorsal metacarpal artery, which branched from the radial artery, communicated with the deep palmar arch through a perforating artery. This perforating artery completed the arch. The radial artery itself ended by supplying the thumb and index finger and had no communication with the deep palmar arch (Figures 3A, 3B).

The deep palmar arch was classified into two patterns based on the branching pattern of the arch. In Pattern 1, the branches of the deep palmar arch, which arose from its convexity, were usually three palmar metacarpal arteries (PMAs). The PMAs were named PMA-1, PMA-2, and PMA-3 from the lateral to the medial side. In this study, 29 (96.7%) hands displayed this pattern, but the course and area of supply of these PMAs varied considerably. Pattern 2 included only one (3.3%) hand in which the deep palmar arch gave only two PMAs.

**Figure 4:**
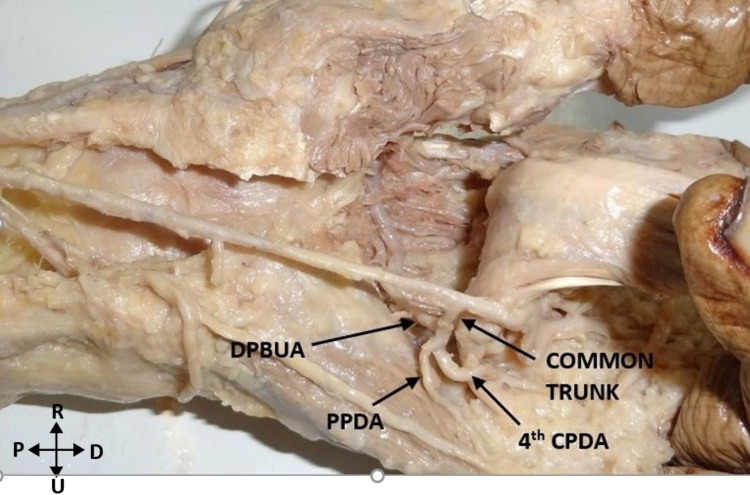
Deep palmar branch arising from the common trunk of origin of fourth CPDA and PPDA to the little finger. DPBUA: deep palmar branch of the ulnar artery; CPDA: common palmar digital artery; PPDA: proper palmar digital artery; P: proximal; D: distal; R: radial; U: ulnar

Two unique variations were observed in this study. The inferior deep branch usually originated directly from the ulnar artery in 24 hands. However, in a left hand, the deep branch arose from the proper palmar digital artery (PPDA) to the little finger. In two other hands, the deep branch arose from the common trunk dividing into the PPDA and the fourth common palmar digital artery (CPDA). This finding was bilateral (Figure 4).

The mean length of the deep palmar arch was 4.1 ± 0.5 cm (4.2 ± 0.47 cm on the right side and 4.0 ± 0.6 cm on the left side). There was no significant difference between the length of the deep palmar arch on the right and left sides (p = 0.617). The mean and standard deviation of the diameters of forming arteries of the deep palmar arch are presented in Table 1. There was no significant difference between the right and left sides in the diameter of the deep palmar branch of the radial artery (p = 0.17) as well as in the diameter of the deep palmar branch of the ulnar artery (p = 0.48). The mean and standard deviation of the diameters of branches of the deep palmar arch are presented in Table 2. There was no significant difference in the diameters of branches of the deep palmar arch between the right and left sides (PMA-1: p = 0.835; PMA-2: p = 0.417; PMA-3: p = 0.26).

**Table 1:** Mean and SD of diameters of the forming arteries of the deep palmar arch. SD: standard deviation; n: number of specimens

Artery	Diameter (in mm) (mean ± SD)		
	Total (both right and left; n = 30)	Right (n = 16)	Left (n = 14)
Deep palmar branch of the radial artery	4.02 ± 0.48	4.20 ± 0.47	4.00 ± 0.56
Deep palmar branch of the ulnar artery	1.90 ± 0.36	1.96 ± 0.37	1.83 ± 0.35

**Table 2:** Mean and SD of diameters of the branches of the deep palmar arch. SD: standard deviation; n: number of specimens; PMA: palmar metacarpal artery

Branch	Diameter (in mm) (mean ± SD)		
	Total (both right and left; n = 30)	Right (n = 16)	Left (n = 14)
PMA-1	1.18 ± 0.35	1.23 ± 0.43	1.11 ± 0.23
PMA-2	1.09 ± 0.28	1.15 ± 0.29	1.04 ± 0.27
PMA-3	1.17 ± 0.31	1.24 ± 0.35	1.09 ± 0.22

## Discussion

The deep palmar arch develops from a plexus at the terminal end of the original axial vessel of the upper limb. The radial artery establishes communication with the deep palmar arch later in development. The anomalies of the deep palmar arch arise mainly during its formation where the forming arteries may originate from a different artery or pursue a different course [8].

The incidence of the complete deep palmar arch in various studies ranges from 76% to 100% [9-13]. In this study, the complete deep palmar arch was found in all cases similar to the studies by Gellman et al. [12] and Loukas et al. [10]. Ikeda et al. noted the complete deep palmar arch in 76.9% of cases [11].

The superior deep branch of the ulnar artery completed the deep palmar arch in 34.5% of cases in the study by Coleman and Anson [9]. Gellman et al. also reported similar findings (33.4%) [12]. On the contrary, Ikeda et al. found a much higher percentage (66%) [11]. The present study observed it in only 10% of cases.

The inferior deep branch of the ulnar artery completed the deep palmar arch in 90% of cases in our study. Gellman et al. and Coleman and Anson observed it in 44.4% and 49% of hands, respectively [9,12]. In contrast, Ikeda et al. found it in only 8.6% of cases. This study did not observe any deep palmar arch completed by both the superior and inferior deep branches of the ulnar artery.

Singh et al. described a variant of the deep palmar arch where a deep branch of the ulnar artery divided into the superior and inferior branches, both of which then anastomosed with the radial artery to complete the deep palmar arch. They found this variant in 8% of cases [14]. The interosseous artery contributed to the formation of the deep palmar arch in previous studies [14,15]. This finding was not observed in this study.

In this study, the radial artery entered through the first intermetacarpal space into the palmar region to complete the deep palmar arch in 96.7% of cases. In a Brazilian study, the deep palmar arch was completed by the radial artery entering through the first intermetacarpal space in 85% of cases [13].

The deep palmar arch completed by the perforating artery communicating with the second dorsal metacarpal artery was noted in 3.3% of cases in this study. Coleman and Anson described this variant as a type of an incomplete deep palmar arch [9]. Ikeda et al. also observed a case where the deep palmar arch was contributed by the perforating artery of the second interosseous space, but the radial artery coming through the first interosseous space was also present. A similar variation has also been reported in monkeys, thereby confirming the phylogenic basis of this variation [11]. Patnaik et al. also found a case in which the deep palmar arch was limited up to the second intermetacarpal space with no contribution further laterally [16].

The PPDA to the little finger gave origin to the deep palmar branch of the ulnar artery in 15% of cases in the study by Olave and Prates [13]. In this study, this variation was found in one case. In addition, a unique variation was found bilaterally in this study in which a common trunk gave rise to the deep palmar branch of the ulnar artery, fourth CPDA, and PPDA.

In this study, the branch from the CPDA or PPDA that completed the deep palmar arch was considered the inferior or distal deep palmar branch of the ulnar artery. This nomenclature is consistent with some previous studies [9,13]. In contrast, another study categorized such an arch as the “radial anastomotic” variant. They argued that because this branch is not a direct branch of the ulnar artery, it should not be considered the deep palmar branch of the ulnar artery [17].

As described in the standard anatomical textbooks [1,18], this study found three palmar metacarpal branches of the deep palmar arch in 96.7% of cases. In 3.3% of cases, only two palmar metacarpal branches were observed. In the study by Coleman and Anson, a minimum of three PMAs were found in a hand. They considered the common trunk giving rise to the princeps pollicis and radialis indicis artery as the first PMA [9]. The present study does not take into account this common trunk. Therefore, in one hand, only two palmar metacarpal arteries were observed.

Although many variant patterns of the palmar arterial arches have been observed in the forming arteries and their branches, anastomosis was found between the radial and ulnar arteries in all cases of the deep palmar arch. This suggests that, from an anatomic perspective, it can be safe to sacrifice the radial artery in procedures such as radial artery harvesting and radial artery flap transfer. Moreover, the radial artery can be used for transradial access in coronary and neuroendovascular interventions. However, it is recommended to use Allen’s test, Doppler ultrasound, and oximetry technique to assess hand circulation before performing any invasive procedures on the radial artery [19,20].

## Conclusions

In this study, anastomosis was found between the radial and ulnar arteries in all cases of the deep palmar arch, implying that all arches were complete. Therefore, the radial artery can be safely sacrificed in procedures such as radial artery harvesting and radial artery flap transfer. Moreover, we observed various patterns of the deep palmar arch and classified them in two ways. This knowledge along with the morphometric data of the arch will undoubtedly help surgeons to perform safe vascular repair surgeries on the hand. However, testing of the hand for vascular insufficiency is recommended before performing any arterial intervention to identify the compromised collateral circulation in the hand.
